# Mechanisms shaping size structure and functional diversity of phytoplankton communities in the ocean

**DOI:** 10.1038/srep08918

**Published:** 2015-03-09

**Authors:** Esteban Acevedo-Trejos, Gunnar Brandt, Jorn Bruggeman, Agostino Merico

**Affiliations:** 1 Systems Ecology, Leibniz Center for Tropical Marine Ecology Fahrenheitstrasse 6, 28359 Bremen, Germany; 2 Jacobs University Bremen Campus Ring 1, 28759 Bremen, Germany; 3 Plymouth Marine Laboratory, Prospect Place, The Hoe, Plymouth, PL1 3DH, United Kingdom

## Abstract

The factors regulating phytoplankton community composition play a crucial role in structuring aquatic food webs. However, consensus is still lacking about the mechanisms underlying the observed biogeographical differences in cell size composition of phytoplankton communities. Here we use a trait-based model to disentangle these mechanisms in two contrasting regions of the Atlantic Ocean. In our model, the phytoplankton community can self-assemble based on a trade-off emerging from relationships between cell size and (1) nutrient uptake, (2) zooplankton grazing, and (3) phytoplankton sinking. Grazing ‘pushes’ the community towards larger cell sizes, whereas nutrient uptake and sinking ‘pull’ the community towards smaller cell sizes. We find that the stable environmental conditions of the tropics strongly balance these forces leading to persistently small cell sizes and reduced size diversity. In contrast, the seasonality of the temperate region causes the community to regularly reorganize via shifts in species composition and to exhibit, on average, bigger cell sizes and higher size diversity than in the tropics. Our results raise the importance of environmental variability as a key structuring mechanism of plankton communities in the ocean and call for a reassessment of the current understanding of phytoplankton diversity patterns across latitudinal gradients.

Understanding ecosystem functioning in relation to species composition and diversity is a central topic in both aquatic and terrestrial ecology^1^,^2^,^3^. Marine phytoplankton, a group of single-celled photosynthetic organisms, are responsible for nearly half of the global primary production on Earth^4^. The size structure of phytoplankton assemblages is a key characteristic of marine ecosystems as it affects the abundance and diversity of organisms in the ocean^5^. The relative abundance of small and large cells of phytoplankton can also influence climate processes and the global biogeochemical cycles of major elements. Most of the biomass produced by small phytoplankton, for example, is thought to be quickly recycled within the euphotic zone, while large phytoplankton cells drive the biological pump by rapidly transporting carbon to the ocean interior^5^. A complex interplay of environmental conditions (e.g. temperature and nutrient concentrations), interspecific relationships (i.e. predation and competition), and dispersal contribute to create heterogeneous patterns of phytoplankton community size structures across different oceanic regions^6^,^7^. Regions with low nutrient concentrations of the tropical and subtropical oceans are dominated by small phytoplankton, whereas regions with high nutrient concentrations support large phytoplankton cells^8^,^9^. Such observations of phytoplankton biogeography have been confirmed by both statistical^10^ and mechanistic^6^ modelling applications at the global ocean scale.

Phytoplankton cell size ranges over several orders of magnitude: from less than 2 *μ*m in equivalent spherical diameter for the picoplankton, 2–20 *μ*m for the nanoplankton, and up to 20–200 *μ*m for the microplankton^11^. Size diversity is therefore another important element in determining ecosystem dynamics, stability, productivity, and nutrient cycling^12^,^13^,^14^. While size diversity represents only one component of functional trait diversity, it is arguably the most important one. As in most organisms, phytoplankton cell size affects numerous other functional traits and crucial physiological and ecological processes, including light absorption, nutrient uptake, sinking, and grazing^11^,^15^,^16^. Quantitative relationships between phytoplankton cell size and such processes can be used to construct mathematical models of phytoplankton community structure. Previous modelling studies based on this view have typically focused on the description of community size composition by resolving many idealised species, size classes, or functional groups and under various levels of physical complexity, from zero-dimensional to ocean circulation models^7^,^17^,^18^,^19^,^20^. These models have resolved the internal cellular physiology of discrete plankton communities and have fostered a functional, trait-based modelling perspective. However, alternative modelling techniques, specifically those that aggregate many species using the “adaptive dynamics” framework^21^,^22^,^23^ promise to provide a strong mechanistic foundation for investigating the structure and the dynamics of phytoplankton communities as “collections of types distributed over trait space”^24^.

Here we present a data-driven, trait-based model to understand the fundamental mechanisms creating compelling differences in community structure and size diversity in two phytoplankton communities of the Atlantic Ocean: temperate and tropical. The phytoplankton community is described in terms of total biomass, mean size, and size variance. By focusing on the dynamics of a key trait such as cell size, this approach leads to reduced model complexity and captures community-aggregate properties of the entire phytoplankton community^21^,^22^,^23^,^25^. The size variance represents a measure of functional diversity^26^,^27^. The simulated phytoplankton community, self-assembles and adapts over time to changing environmental conditions and subject to a trade-off. The trade-off emerges from fundamental relationships imposed between phytoplankton cell size and: (1) phytoplankton nutrient uptake, (2) zooplankton grazing, and (3) phytoplankton sinking. Being based on fundamental principles of phytoplankton trait ecology, our model provides a concise and quantitative framework to study structure and functional diversity of phytoplankton communities in regions of the oceans with contrasting environmental regimes (Figure 1).

## Results

Despite its simplicity in terms of number of state variables and number of parameters, the model correctly reproduces the typical and most important ecological features of the two contrasting regions (see Supplementary Text S1 and Supplementary Figure S2). The imbalances between top-down and bottom-up processes regulate the dynamics of phytoplankton communities, which results in the observed high biomasses in the temperate region (See Supplementary Figure S3). These two forces are better balanced in the tropics leading to an almost constant biomass concentration throughout the year (See Supplementary Figure S3).

Moreover, the model produces significantly different mean cell size dynamics in the two contrasting regions (Fig. 2). In the temperate, mean cell size follows a seasonal pattern with a maximum (≈3.2 Log *μ*m Equivalent Spherical Diameter or ESD) in spring and a minimum (≈2.2 Log *μ*m ESD) stretching from summer to autumn. Although the mean cell size remains within the upper nanoplankton range throughout the year, a fraction of the community can exceed 3.7 Log *μ*m, as indicated by the standard deviation (shaded area in Fig. 2A). In the tropics, mean cell size is notably lower than in the temperate (≈Log1.3 *μ*m ESD), relatively constant (Fig. 2B), and consistently confined to the lower nanoplankton size class throughout the year (shaded area in Fig. 2B). Our model results are consistent in shape and magnitude with High Performance Liquid Chromatography (HPLC) data (coloured dots at the centre of each Phytoplankton Size Class or PSC in Fig. 2), which clearly reveal the dominance of nanoplankton in the temperate region (dark green dots in Fig. 2A) and the importance of picoplankton and nanoplankton in the tropics (dark green dots in Fig. 2B). These data represent a strong constraint to the model and, therefore, to the feasibility of mechanisms that can explain community size structure and functional diversity in the two regions.

Community mean cell size responds to the changing environment, which selects different trait values according to a trade-off emerging from the three different size-scaling processes considered in the model (Equations 11, 12 and 13). Specifically, the dynamics of mean cell size in the temperate setup is influenced by the interplay of zooplankton grazing and nutrient uptake (Fig. 2C) throughout the seasons. In winter the influence of these two processes is much smaller than during the rest of the year, as consequence of a nutrient replete environment with a low zooplankton concentration (Fig. 2C). The impact of these top-down and bottom-up processes increases during spring when zooplankton grazing becomes slightly more important than phytoplankton nutrient uptake. Grazing ‘pushes’ the community towards larger cell sizes, whereas nutrient uptake ‘pulls’ the community towards smaller cell sizes. In contrast, the tropical setup reveals a balance between grazing and nutrient uptake, which results in a constant mean cell size throughout the year (Fig. 2D). In both setups sinking plays either a minor (in the temperate) or an irrelevant (in the tropics) role (green area in Fig. 2C and 2D).

The model also simulates the size diversity of the two contrasting communities. This diversity metric is represented by the size variance (Equation 7) and is indicated in our results as one standard deviation around the mean cell size (shaded area in Fig. 2A and 2B). On average, the temperate region shows a 1.3 times higher size diversity than the tropical region (the average annual variance is 0.21 [Log *μ*m ESD]^2^in the temperate region compared to 0.16 [Log *μ*m ESD]^2^ in the tropical region). Size diversity is also positively correlated with mean cell size and biomass, especially reflected by the seasonal signal in the temperate region (Fig. 3). Thus, the community dominated by larger cells (i.e. the temperate community) reaches higher size diversity and accumulates more biomass than the community dominated by smaller cells (i.e. the tropical community). To verify that these results are independent from the model parameterisation, we varied all model parameters by ±50%, and quantified the deviations in the diversity ratio between the two regions (i.e. deviations from the 1.3 ratio of the standard runs). None of these substantial changes in the parameterization produced appreciable alterations of our results in the sense that size diversity in the temperate region remained always higher than size diversity in the tropical region (i.e. their ratio is always above 1, Fig. 4).

To disentangle the relative contribution of the different environmental factors to the emergence of the contrasting community size structures and size diversities, we tested the effects of environmental forcing. Specifically, we interchanged the forcing variables, one at a time, between the regions and quantified how much the specific setup changed from the standard model run (Supplementary Figure S4 and S5). This experiment revealed that the largest changes in community size composition and diversity are caused by those environmental variables that control the availability of nutrients. These variables are the mixed layer depth (MLD) and the nutrient concentration below the mixed-layer (N_0_). The temperate community shifts from larger to smaller cell sizes and from higher to lower size diversity when it experiences the MLD or the N_0_ of the tropical region (Supplementary Figures S4 and S5). The tropical community, instead, shifts from smaller to larger cell sizes and from lower to higher size diversity when it is exposed to the MLD or to the N_0_ of the temperate region (Supplementary Figures S4 and S5). The changes of PAR and SST have a positive effect on the mean cell size in both regions, but this alteration did not have a major effect on size diversity (Supplementary Figures S4 and S5).

In addition, we assessed the effects of changing environmental conditions on the relative importance of the three size-scaling processes. These processes are more sensitive to changes in nutrient availability than to changes in any other forcing (Supplementary Figure S6). For example, the stable MLD of the tropical region reduces the seasonal imbalances between nutrient uptake and grazing when it is applied to the temperate region so that shifts in mean cell size and size diversity become less pronounced (cf. the effect of the tropical MLD in the temperate setup, Supplementary Figures S4, S5, and S6). Exposing the tropical region to the MLD of the temperate region, instead, breaks the balance between nutrient uptake and grazing and leads to pronounced seasonal variations in mean cell size and size diversity (cf. the effect of the temperate MLD in the tropical setup, Supplementary Figures S4, S5, and S6). Tropical PAR and SST tend to smooth out the effects of the size-scaling processes in the temperate setup, while temperate PAR and SST have a minor effect in the tropical setup (cf. Fig. 2C–2D and Supplementary Figure S6). Exchanging the deep-layer nutrient concentration N_0_ in the two setups results in decreasing mean cell size and size diversity in the temperate region and increasing mean cell size and size diversity in the tropical region (Supplementary Figures S4 and S5), but does not considerably change the dynamics of nutrient uptake and zooplankton grazing throughout the year (cf. Fig. 2C–2D and Supplementary Figure S6).

Total phytoplankton biomass P_T_, mean cell size 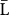, and size variance V are relatively insensitive to changes in model parameters (Table 1). None of these three macroscopic properties vary more than ±30% when most of the parameter values are changed by ±25% (Supplementary Figure S7), which leads to small qualitative deviations of the seasonal signal and of overall regional difference. The only exception is for the parameters *μ*_Z_ and *α*_G_ that control grazing pressure, which cause appreciable changes in P_T_ and 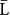. All macroscopic properties are very robust (with a maximum of 18% shift in size variance) with respect to changes in the immigration rate (*δ*_I_) and the size variance of the immigrating community (V_0_) (Supplementary Figure S7).

## Discussion

One way to understand marine communities is to think of them as an ensemble of highly interconnected groups of organisms, each with its own characteristic features. This perspective, however, requires to account for a considerable number of details particularly when studying macroscopic properties such as mean trait or trait diversity. According to Complex Adaptive Systems theory, these properties are generally insensitive to the characteristics of a particular species or even group of species, although they emerge from the aggregate dynamics of a large number of interactions among them^28^. To understand the broad patterns of phytoplankton species composition and abundance and to quantify to which degree are these patterns determined by environmental conditions, we adopted an approach that allowed us to describe the time variation of macroecological properties with simple but powerful rules (i.e. functions trading-off competitive abilities) driving change. In contrast to previous studies^6^,^7^,^17^,^18^,^19^,^20^, which provide useful insights into the mechanisms driving community structure and diversity at local and global scales, albeit without direct comparison to *in situ* trait distributions, we focused on only two contrasting regions with a good availability of both environmental forcing and phytoplankton cell size data.

In agreement with present day understanding of marine phytoplankton ecology^8^,^29^,^30^,^31^, our model shows that the community of the temperate region exhibits a more pronounced seasonality than the community of the tropical region (Supplementary Material S1, S2 and S3). In the temperate region, changes in community size composition are characterised by a seasonal shift from large mean cell size in spring, within the range of nano- and microplankton, to small mean cell size in autumn, within the nanoplankton range (Fig. 2A). In contrast, small mean cell sizes, confined to the lower level of the nanoplankton range, dominate the tropical region throughout the year (Fig. 2B). These simulated patterns are consistent with *in situ* PSC observations (green dots in Fig. 2A and 2B), with Continuous Plankton Recorder data^31^, fall within the ranges of previous observations made in the Atlantic Ocean^32^,^33^, and compare well to phytoplankton size class estimates derived from satellite imagery^34^. Our sensitivity analyses highlight the role of nutrient availability in shaping the resulting size patterns (Supplementary Figure S4, S5 and S6) and the general robustness of the model results to changes in parameter values (Supplementary Figure S7). These observations are consistent with a modelling study focused on diatoms that highlighted the importance of different nutrient regimes and disturbances for regulating the community size structure of this relevant phytoplankton group^35^.

We also find that the two regions differ with respect to size diversity (Fig. 3). Specifically, the simulated size diversity in the temperate region is 1.3 times higher than in the tropical region. A globally resolved model of marine phytoplankton^19^ produced, in line with the latitudinal diversity gradient tenet, a decline in species richness with increasing latitude. Our work challenges this standing viewpoint. Recent studies^36^,^37^,^38^ have casted serious doubts on the validity of conventional sampling techniques for estimating the diversity of marine microbial communities. In line with our results, new corrected estimates of species richness^36^ point to a higher number of species in the temperate coastal ecosystem of Ría de Vigo as compared to nutrient poor regions of the tropics and subtropics. Such studies constitute a lively and developing area of research and our results provide further evidence on the importance of reassessing current understanding on diversity patterns with new and more accurate sampling techniques.

The size diversity captured by our model is, similarly to species richness, only one component of biodiversity^27^. Analysing diversity patterns that use different metrics of diversity poses challenges that are beyond the scope of our study. Nevertheless, our results shed new light on the coupled dynamics of phytoplankton biomass, cell size, and size diversity (Fig. 3). We find that the environmental stability of the tropics dampens potential seasonal fluctuations in phytoplankton mean cell size thus producing lower size diversity there than in the temperate region (Fig. 2 and 3). The stable environmental conditions produce balanced and strongly opposing top-down and bottom-up forces, respectively zooplankton grazing and nutrient uptake, leading to reduced size diversity (Fig. 2 and 3). In contrast, the seasonally pulsing environment of the temperate region produces periods during which nutrient uptake dominates, thus pushing mean cell size and size variance to higher values (spring), and other periods during which grazing dominates, thus reducing mean cell size and size variance to smaller values (autumn). The environmental seasonality, therefore, drives such ‘readjustments’ in community structure via shifts in species composition and provides adaptive capacity to the system in terms of increased functional diversity (Fig. 2 and 3). These emergent properties are robust features of our model results as an in-depth sensitivity analysis confirms (Fig. 4). In addition, our sensitivity analyses show that size composition and diversity of both tropical and temperate communities respond most sensitively to nutrient availability (Supplementary Figures S4, S5 and S6).

Models are only approximations of reality and, as in all modelling efforts, our study is based on a number of simplifications. Sinking, for example, plays only a marginal role in shaping community structure and size diversity of both regions probably because of the simplified description of the mixed layer depth dynamics. Our model focuses on the temporal dynamics of a single trait while organisms possess a myriad of them. In addition, by aggregating collections of species types over trait space, our model cannot resolve single species or functional groups nor can it differentiate between genetically distinct but morphologically similar organisms (cryptic species) or genetically similar but morphologically distinct organisms (ecotypes). In common to previous models^22^,^25^, here size diversity is sustained by a positive source of variance, immigration, which accounts for unresolved processes such as spatiotemporal heterogeneities, interspecific interactions, or the presence of resting stages. Also, other specific processes such as size-selective grazing are not considered here. Nevertheless, our approach provides evidence that a simple and mechanistically sound trait-based model in combination with realistic environmental settings presents a powerful tool for explaining the broad patterns of phytoplankton community composition in environmentally contrasting regions of the ocean.

The importance of environmental variability (represented in our model by changes in MLD, SST, PAR and N_0_) has already been recognized as a key structuring mechanism of diatom communities in marine and freshwater systems^25^,^35^,^39^. Our work extends these findings (1) by considering a whole phytoplankton community not just a limited number of functional groups and (2) by providing a simple quantitative framework that describes the coupled dynamics of three fundamental macroecological properties of phytoplankton communities, namely biomass, means size, and size diversity.

## Methods

### Model description

Our model is based on the typical physical scheme of the upper ocean with mixed layer dynamics^40^, see table 1 for a list of parameter names, values, and units. The seasonal dynamics of the mixed layer depth (MLD) is given by the forcing function M(t) with the change of the MLD denoted by h(t) = dM(t)/dt and t the time in days. Material exchange between the upper mixed layer and the bottom layer are described by vertical turbulent diffusion and by entrainment or detrainment caused by deepening or shallowing of the upper mixed layer^40^. Following Evans & Parslow^41^, we use h^+^(t) = *max*[h(t), 0] to account for the effects of entrainment and detrainment. Zooplankton is considered capable of maintaining themselves within the upper mixed layer, therefore, we define the dilution and the concentration of zooplankton resulting from changes in MLD as h(t). Diffusive mixing across the thermocline, *κ*, is represented by means of a constant factor. The whole diffusion term is, thus, given by
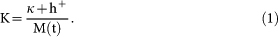
The model follows the adaptive dynamics approach^21^,^22^,^23^. This approach describes the distribution and dynamics of a characteristic trait of an entire community and, by this means, reduces complexity^23^. A moment closure technique is applied to approximate the community dynamics with three macroscopic properties: (a) total biomass, (b) mean trait, and (c) trait variance, the latter reflecting the functional diversity of the community. We choose cell size (S in *μ*m Equivalent Spherical Diameter, ESD) as the characteristic trait of the phytoplankton community. The distribution of phytoplankton cell size is known to follow a log-normal distribution^42^, therefore, following the standard approach^25^,^43^,^44^, we log-transform the size trait, L = log(S). The changes in total community biomass (P) over time t are given by:
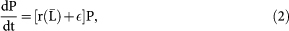
where 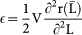 denotes higher order moments resulting from the moment closure technique^22^,^23^. 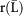 is the net growth rate of the total phytoplankton biomass, i.e. gains minus losses in P.
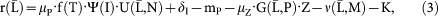
where *μ*_P_ is the maximum specific growth rate at temperature T = 0°C and f(T) = e^0.063·T^ represents Eppley's formulation of temperature-dependent growth. The light limitation term, Ψ(I), integrates the photosynthetically active radiation (PAR) *I* through the mixed layer by using Steele's formulation:
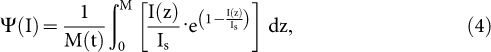
where I_s_ is the light level at which photosynthesis saturates and *I*(*z*) is the PAR at depth *z*. The exponential decay of light with depth is computed according to the Beer-Lambert law with a generic extinction coefficient k_w_
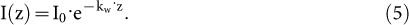
The nutrient-limited uptake term 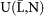 depends on the nutrient concentration and scales with phytoplankton cell size (Equation 11). *δ*_I_ accounts for the dispersal rate of phytoplankton (i.e. immigration) from adjacent patches^22^ into the considered community and m_P_ accounts for all possible phytoplankton losses other than grazing and mixing (K). The term 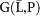 denotes zooplankton grazing, which is a function of phytoplankton cell size (Equation 12). Finally, the term 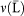 represents the size-dependent sinking (Equation 13).

Temporal change in mean cell size is described by the adaptive dynamics equation
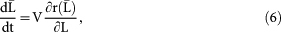
where V is the variance of the log size or functional size diversity of the community. The temporal evolution of the variance (V) is given by
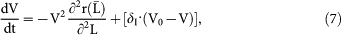
where V_0_ is a source of size variance from an immigrating community outside the modelled region.

Differential equations for nutrients (N), zooplankton (Z), and detritus (D) complete the model system:
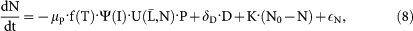

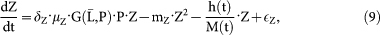

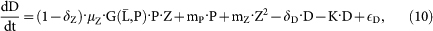
where 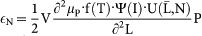, 
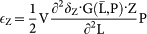
, 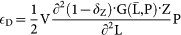. These terms account for higher order moments resulting from the moment closure technique.

The dynamics of our size-based model is constrained by a trade-off emerging from fundamental relationships between phytoplankton cell size and (1) phytoplankton nutrient uptake, (2) zooplankton grazing, and (3) phytoplankton sinking. Arguably, a number of other size-dependent physiological and life history processes could be considered in the model^11^. Our approach, however, was to develop a model simple enough to capture the phytoplankton community composition by accounting for the minimal number of important top-down and bottom-up regulatory mechanisms. The following sections provide further details on the size relationships considered in the model. Note that we use typical power laws for the allometric relationships; however, when we log-transform size, the allometric dependency on size becomes an exponential dependency on log size.

### Phytoplankton nutrient uptake

Nutrient uptake U is determined by a Michaelis-Menten type formulation with a half-saturation constant K_N_ that scales allometrically with the phytoplankton cell size L,
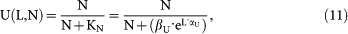
where 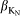 and 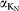 are, respectively, intercept and slope of the K_N_ allometric function. This empirical relationship is based on observations of different phytoplankton groups^45^, with the regression parameters rescaled from cell volume to ESD. Plotting equation 11 for a range of different values for N and L reveals that smaller phytoplankton cells have distinctively higher nutrient uptake rates than larger ones over a broad range of nutrient concentrations (Supplementary Figure S7A), which is consistent with the current understanding of phytoplankton community structure under different nutrient regimes^15^,^16^,^46^. This advantage of small cells is most pronounced at low nutrient levels and reduces with increasing nutrient concentrations.

### Zooplankton grazing

The second size-scaling process we considered in the model is zooplankton grazing. Both theoretical and experimental evidence suggests the existence of an eco-evolutionary trade-off that selects for smaller phytoplankton cells under increasing grazing pressure^23^,^47^,^48^. We therefore consider a simple grazing formulation based on a Michaelis-Menten type function,
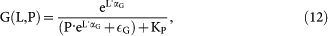
where *α*_G_ is the slope for allometric grazer preference, K_P_ is the half saturation constant for grazing and 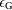 is a correction term for higher order moments, i.e. 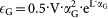. This formulation considers in the denominator a second-order approximation of the weighted integral of phytoplankton biomass (i.e. community integral of 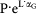). As expected, this formulation leads to diminishing grazing pressure with increasing phytoplankton cell size (Supplementary Figure S7B) and, thus, counteracts the competitive advantage of small cells under low nutrient concentrations.

### Phytoplankton sinking

Finally, we consider a relationship between phytoplankton cell size and sinking velocity based on Stokes' law, which predicts increasing sinking velocities with increasing cell size^49^
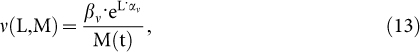
where the constants *α_ν_* and *β_ν_* are the parameters of the allometric function proposed by Ref. 49, which are modified here to reveal units in meters per day. This relationship favours smaller phytoplankton cells over larger ones (Supplementary Figure S7C), because a higher sinking speed translates into a higher export from the mixed layer, i.e. into a higher mortality for large cells.

### Adaptive dynamics framework

Our modelling approach is developed within the conceptually broad context of Complex Adaptive Systems (CAS) theory^28^. More specifically, the adaptive dynamics model we formulated is rooted in quantitative genetics theory, which typically describes the long-term evolutionary dynamics of quantitative traits as driven by mutation and selection under some strict assumptions^50^,^51^,^52^. Note, however, that adaptive dynamics is not limited to mutations^22^,^53^,^54^ and that size diversity in our model is described by the standing variance of the trait distribution, which is assumed to be unimodal. The model therefore cannot account for the emergence of multimodality. However, in Supplementary Figure S9 we show that the second derivative of the net specific growth rate is, in both regions, always negative, indicating that the community dynamics generated by the model always reduces variance and that multimodality is unlikely to occur in our set up.

### Phytoplankton immigration

Because of competitive exclusion^55^, modelled diversity tends to collapse over time both in adaptive dynamics models^22^,^23^ and in models that explicitly resolve many different species^6^,^56^. This causes the phytoplankton community to ‘loose’ adaptive capacity over time (i.e. 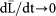 in Equation 6). The problem is typically circumvented by including a source of positive variance, typically immigration^25^. Following Terseleer et al.^25^, we treat immigration as a density-dependent process. Preliminary experiments with our model showed that introducing trait values at a constant rate predefines higher diversity at lower biomass and vice versa. Instead, our density-dependent immigration (indicated by the last term in equation 2) predicts that communities located in regions with higher biomass have also higher immigration rates, reflecting the assumption that adjacent regions are similar (in terms of community structure) to the simulated region of interest. Recent data analysis of phytoplankton diversity patterns in the Atlantic Ocean support this assumption^57^.

In addition, we assume that the cell sizes of the immigrating phytoplankton are equal to the prevailing mean cell size of the residents (i.e. 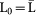) to avoid any systematic bias on the mean trait of the resident community. Therefore, we assume that the immigrating community has been exposed to the same selection pressures (i.e. same fitness gradient, causing to converge to the same trait values) as the resident community. These assumptions play an important role in sustaining the functional diversity of the considered community and are well recognized features of the complex adaptive systems approach^22^,^25^,^28^,^58^.

Given the uncertainties involved in constraining the rates of immigration in the ocean we considered this parameter (*δ*_I_) as free and allowed it to vary within a relatively narrow range in order to fit the size data (see Table 1). The small rate we obtained for the standard run (0.008 d^−1^) ensures that immigration does not affect the results substantially by superimposing externally forced dynamics. This is also supported by the results of the sensitivity analysis (cf. Supplementary Figure S7).

### Model setups

**Figure 1:**
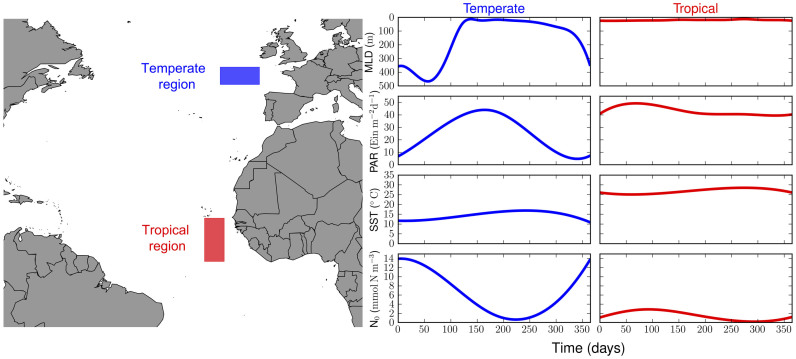
Model setups. The left panel shows the geographical locations of the two considered regions: temperate and tropical. The panels on the right show the temporal changes of the environmental variables, namely mixed-layer depth (MLD), photosynthetically active radiation (PAR), sea surface temperature (SST), and nutrient concentration below the MLD (N_0_). The map was generated using R v.3.1.2 (The R Foundation for Statistical Computing, Vienna, Austria).

**Figure 2:**
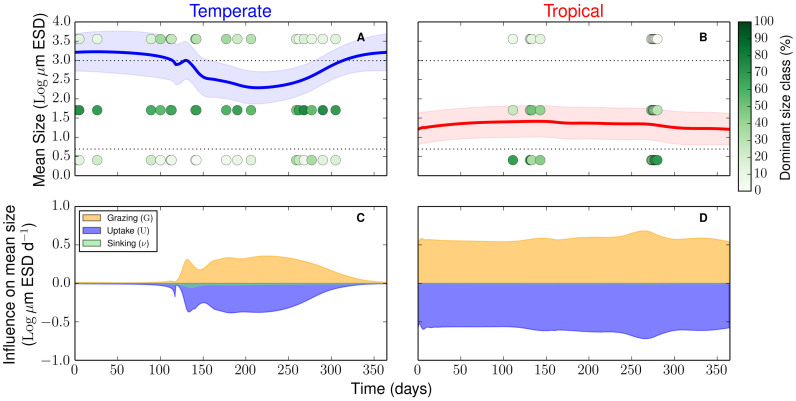
Mean cell size dynamics in the temperate and tropical regions. The shaded areas indicate one standard deviation calculated from the size variance (or functional diversity, equation 7). The dashed lines mark the limits of the phytoplankton size classes (PSC), which are picoplankton, nanoplankton, and microplankton. The dots represent PSC observations reconstructed from High Performance Liquid Chromatography (HPLC) data^59^,^60^. The green colour scale represents the relative dominance of each PSC in percent. The bottom panels show the influence of the size-scaling processes on mean cell size (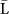) in the temperate (C) and in the tropical (D) regions. The orange area represents zooplankton grazing (Equation 12), the blue area represents nutrient uptake (Equation 11), and the green area represents phytoplankton sinking (Equation 13). The positive values of zooplankton grazing indicate that this process drives the community composition towards larger sizes, while nutrient uptake and phytoplankton sinking drive the community towards smaller sizes.

**Figure 3:**
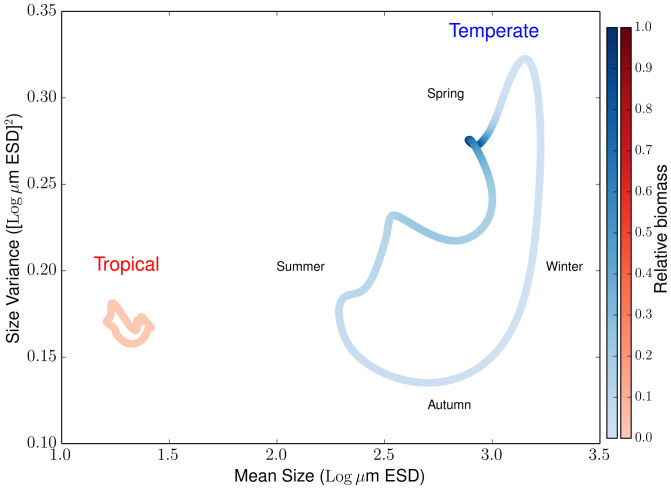
Phase plane of the mean cell size and size variance, the latter reflecting the functional size diversity. The lines corresponds to a seasonal changes of the two state variables for the tropical and temperate regions. Changes in colour tonalities reflect changes in the relative biomass of each region.

**Figure 4:**
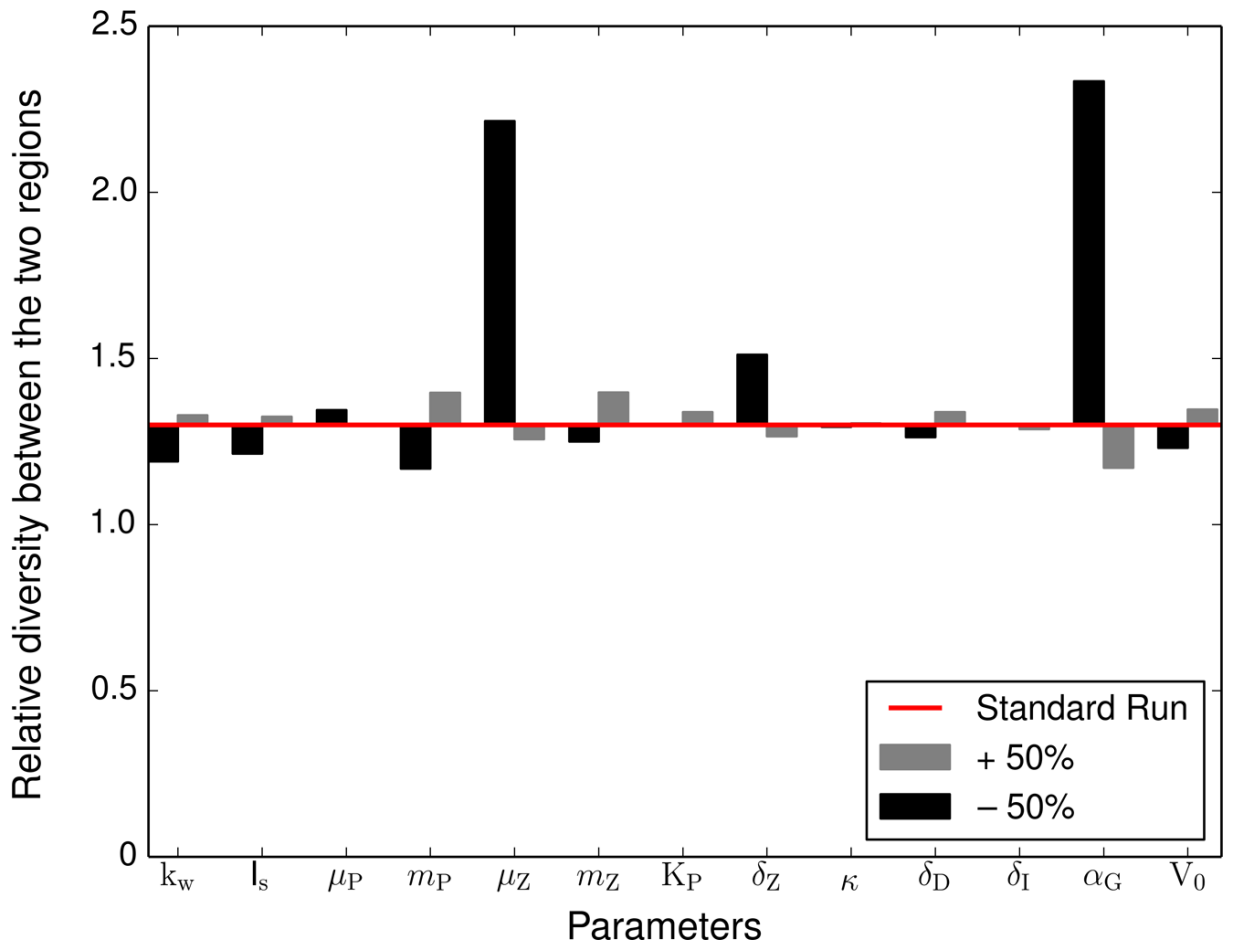
Sensitivity of the diversity ratio between the temperate and tropics to changes in model parameters. The red line marks the diversity ratio between the two regions obtained with the standard run (≈1.3). The bars show the changes of this ratio for alterations in the model parameters by +50% (grey bars) or −50% (black bars). The names and reference values of the parameters are listed in Table 1.

For comparison, our model is applied to two regions of the Atlantic Ocean with contrasting environmental conditions (Fig. 1), namely a temperate and a tropical region. The temperate model setup is characterized by pronounced seasonal changes in mixed layer depth (MLD), photosynthetically active radiation (PAR), sea surface temperature (SST), and nutrient concentration below the mixed-layer (N_0_) (Fig. 1), whereas the tropical model setup shows a lack of seasonality (Fig. 1). Besides these differences, the two model setups are identical in terms of parameterisation and model structure. We assume that conditions are homogeneous within the chosen rectangular regions and that the model results are representative of the whole rectangular areas.

In a spin-up phase of four years the model reaches quickly the steady-state and we then consider the last year of a five-year simulation for our analyses and discussions.

### Sensitivity Analysis

The model contains only 13 parameters (Table 1), it is therefore practical in our case to examine the effects that changes in parameter values have on model results. To this aim, we formulate a sensitivity index S that accounts for relative changes in model results as follows:
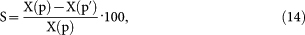
where X(p) is the value of the state variable obtained with the standard parameter value p and X(p′) is the value of the state variable obtained with parameter value p′ = p ± 50%, for the sensitivity of the relative diversity between the two regions, or p′ = p ± 25%, for the sensitivity of the annual mean of P, 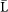 and V.

### Locations and data

The tropical and temperate locations and their areal extent were selected according to the following compromising principle: (1) availability of size data and (2) homogeneity of environmental conditions. Localized effects such as coastal upwelling can therefore be excluded.

The two model setups were forced with climatological data of MLD from the World Ocean Atlas 1994 (WOA94), PAR and SST from the Moderate Resolution Imaging Spectroradiometer (MODIS), and N_0_ from the World Ocean Atlas 2009 (WOA09). All these variables were spatially averaged within the chosen rectangular areas.

To constrain the temporal evolution of the mean cell size predicted by our model we compiled High Performance Liquid Chromatography (HPLC) measurements falling into our two regions of interest from the Atlantic Meridional Transect programme^59^ and from the Geochemistry, Phytoplankton and Color of the Ocean project^60^. These measurements quantify the concentration of pigments in each sample, which provides an indication of the community composition of photosynthetic organisms^61^. Each phytoplankton group has a characteristic set of pigments that allows for a differentiation on the level of functional groups or Phytoplankton Size Classes (PSCs)^62^. We reconstructed the PSCs from diagnostic pigments following^61^ and then calculated the relative composition of each PSC as the sum of pigments in each PSC divided by the total sum of the diagnostic pigments. The resulting size classes are picoplankton (0.2–2 *μ*m), nanoplankton (2–20 *μ*m), and microplankton (above 20 *μ*m). The differences observed in terms of HPLC measurements are consistent with *in-situ*^8^ and satellite^34^,^62^ observations.

## Supplementary Material

Supplementary Information Supplementary Material

## Figures and Tables

**Table 1:** Model parameters. The parameters with source “this study” were considered as free parameters, therefore, manually chosen, and allowed to vary in order to obtain a better model to data fit (i.e. a good approximation of model results to all data on concentration of nutrients, phytoplankton biomass, and mean size for both regions)

Name	Symbol(Units)	Value	Source
P growth rate	*μ*_P_ (d^−1^)	1.4	[63]
P mortality rate	m_P_ (d^−1^)	0.05	[40]
P immigration rate	*δ*_I_ (d^−1^)	0.008	this study
Size variance of immigrating P	V_0_ (Log [*μ*m ESD]^2^)	0.58	this study
Z growth rate	*μ*_Z_ (d^−1^)	1.1	[40]
Z mortality rate	m_Z_ (d^−1^)	0.3	[63]
P assimilation coefficient	*δ*_Z_ (-)	0.3	[63]
P half-saturation	K*_P_* (mmol N m^−3^ *μ*m^−1^ ESD)	0.1	this study
Cross-thermocline mixing	*κ* (m·d^−1^)	0.01	[64]
Mineralization rate	*δ*_D_ (d^−1^)	0.1	[40]
Light attenuation constant	k_w_ (m^−1^)	0.1	[63]
Optimum irradiance	I*_s_* (E m^−2^d^−1^)	30	this study
Intercept of the K*_N_* allometric function	*β_U_*	0.14257	[45]
Slope of the K*_N_* allometric function	*α_U_*	0.81	[45]
Intercept of the *ν* allometric function	*β_ν_*	0.01989	[49]
Slope of the *ν* allometric function	*α_ν_*	1.17	[49]
Slope for allometric grazer preference	*α_G_*	−0.75	this study
