# Supplementary material for: Mechanisms shaping size structure and functional diversity of phytoplankton communities in the ocean
Source: Sci Rep. 2015 Mar 9;5:8918. doi: 10.1038/srep08918 (PMC5390085; doi:10.1038/srep08918)
Supplement: Supplementary Information — Supplementary Material [file srep08918-s1.pdf]

Mechanisms shaping size structure and functional diversity of phytoplankton communities in the ocean. Acevedo-Trejos, E., Brandt, G., Bruggeman, J. and Merico, A.

Supplementary Material

Supplementary Text S1

The same model, in terms of structure and parameterization (see Methods section), was applied to a tropical and a temperate region of the Atlantic Ocean (Fig. 1). The only differences between the two model applications were the specific environmental conditions (i.e. the forcing) characterizing each region. As expected, the model results in the temperate region exhibit a pronounced seasonality (Supplementary Figure S2) with high nutrient concentrations and low phytoplankton biomass during winter (Supplementary Figures S2A and S2C) and a biomass build up typical of a phytoplankton bloom event with the associated nutrient decrease in spring (Supplementary Figures S2A and S2C). Nutrient concentrations and phytoplankton biomass remain low during summer (Supplementary Figures S2A and S2C). The seasonal overturn of the water column restocks the nutrient pool in autumn (Supplementary Figures S2A and S2C). Consistently, also zooplankton and detritus show a pronounced seasonality (Supplementary Figures S2E S2G). In contrast, all variables are constant in the tropical region and relatively low throughout the year (Supplementary Figure S2B, S2D, S2F, and S2H). The simulated temporal evolutions of nutrient concentrations and phytoplankton biomasses in the two regions compare well with the available observations (Supplementary Figures S2A to S2D). Hence, despite its simplicity in terms of number of state variables and number of parameters, the model is able to correctly reproduce the typical and most important ecological features of the two contrasting regions.

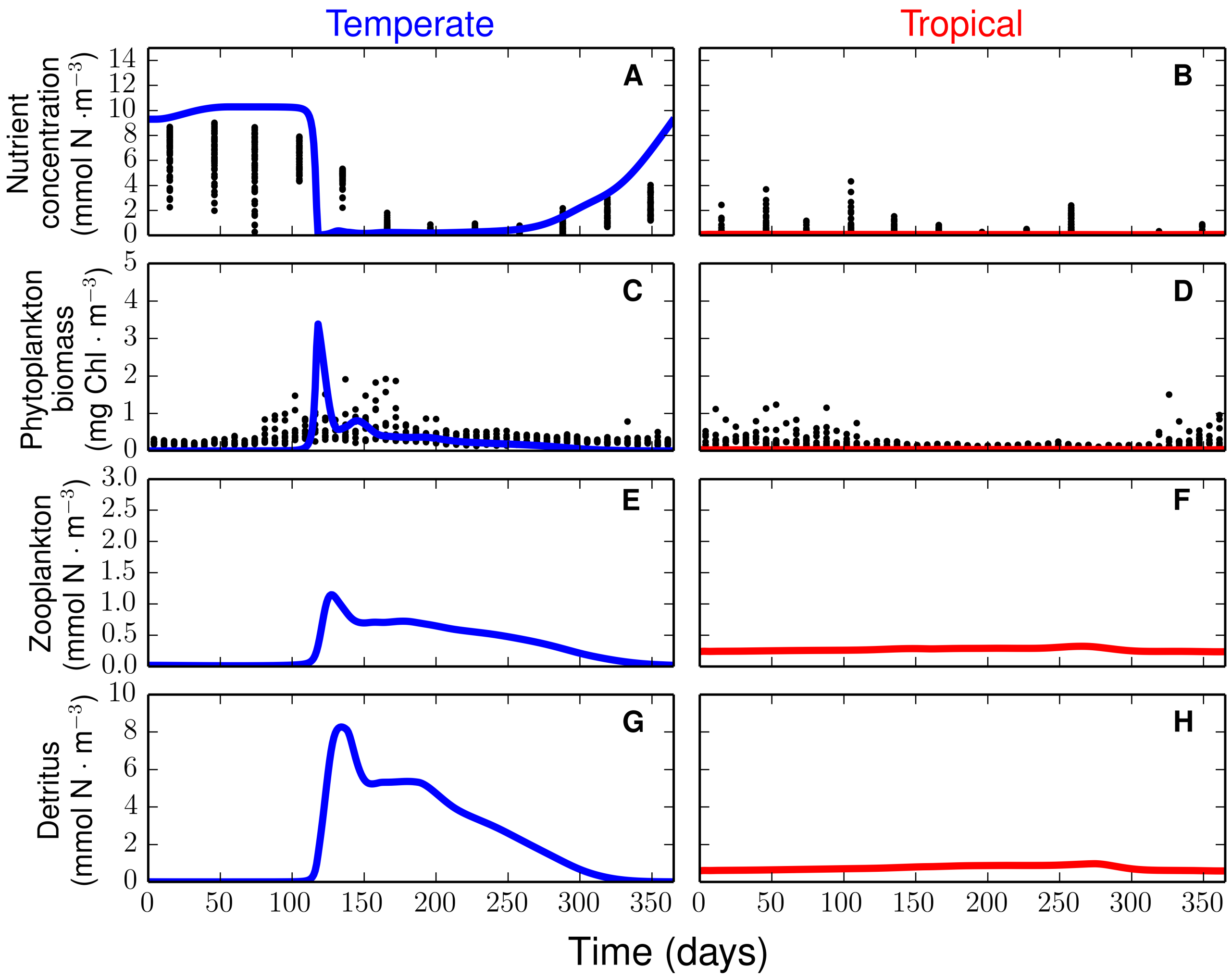

Supplementary Figure S2. Temporal changes of the state variables obtained with the temperate (left column, blue lines) and tropical (right column, red line) setups. The black dots correspond to surface nutrient observations from the World Ocean Atlas (WOA2009) (A and B) and to chlorophyll observations from MODIS (C and D).

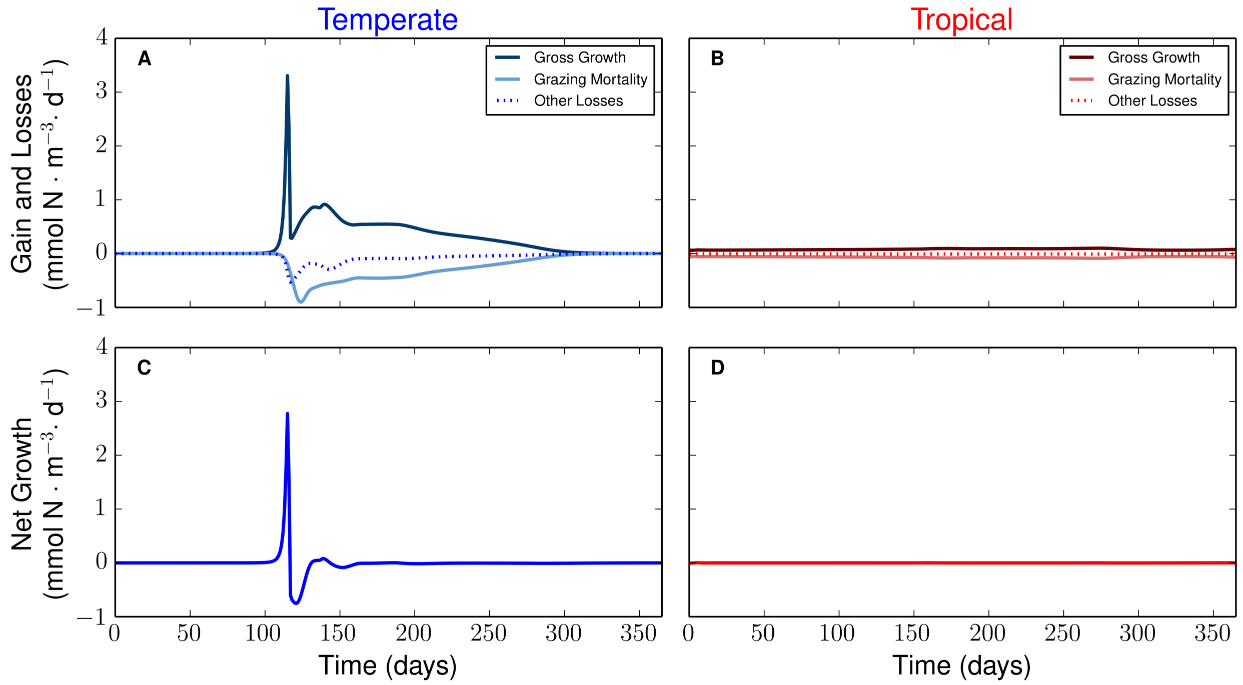

Supplementary Figure S3. Gains and losses of phytoplankton growth in temperate (A) and tropical (B) regions by processes. Panels C and D show the resulting net growth considering all processes affecting phytoplankton biomass.

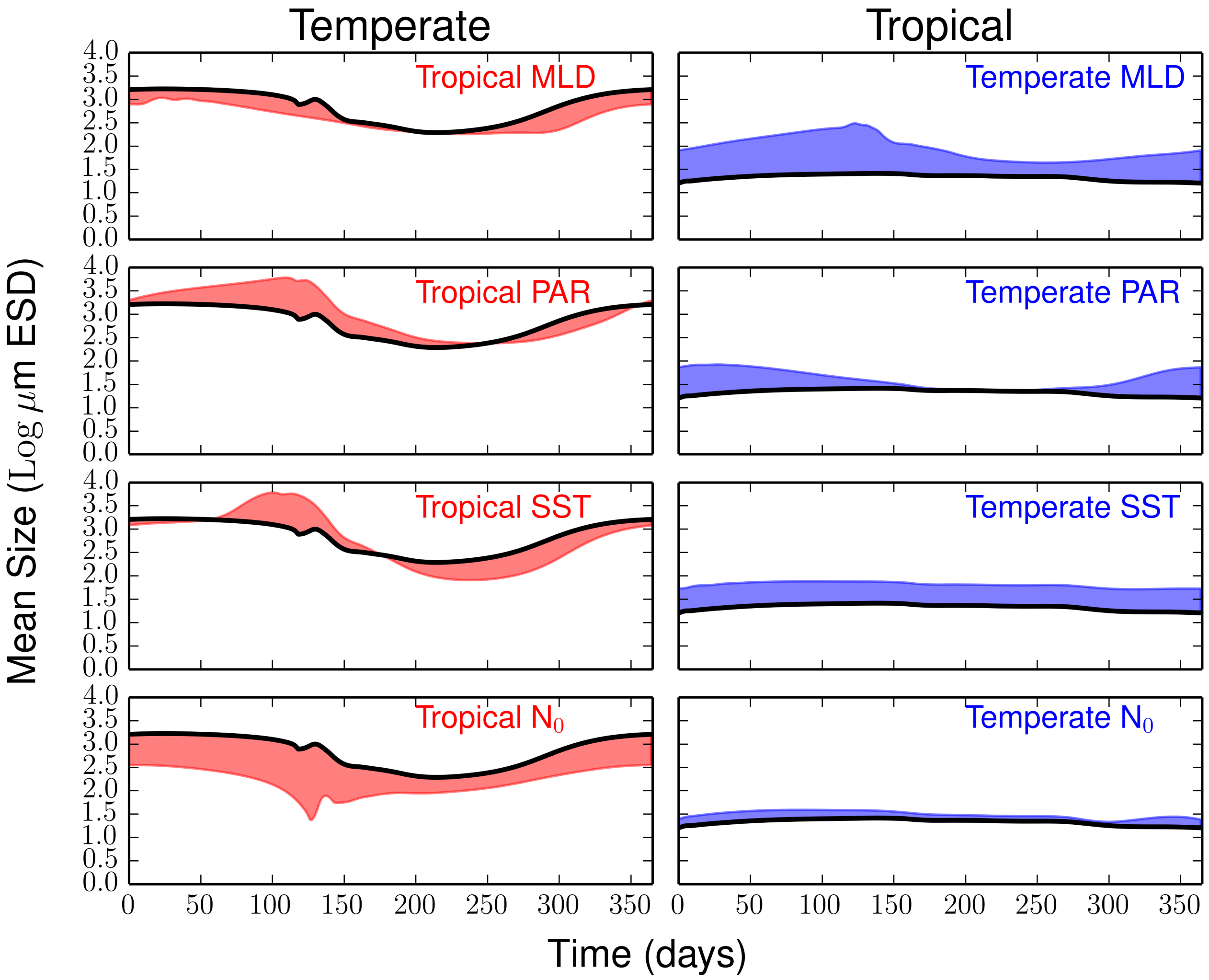

Supplementary Figure S4. Sensitivity of community size composition to changes in environmental forcing. The black lines are the mean sizes obtained with the standard runs, as shown in Fig. 4. The red areas in the temperate setup (left panels) show the effects on the mean size in the temperate region when one of its environmental conditions is changed with one of the tropical region. The blue areas in the tropical setup (right panels) show the effects on the mean size in the tropical region when one of its environmental conditions is changed with one of the temperate region.

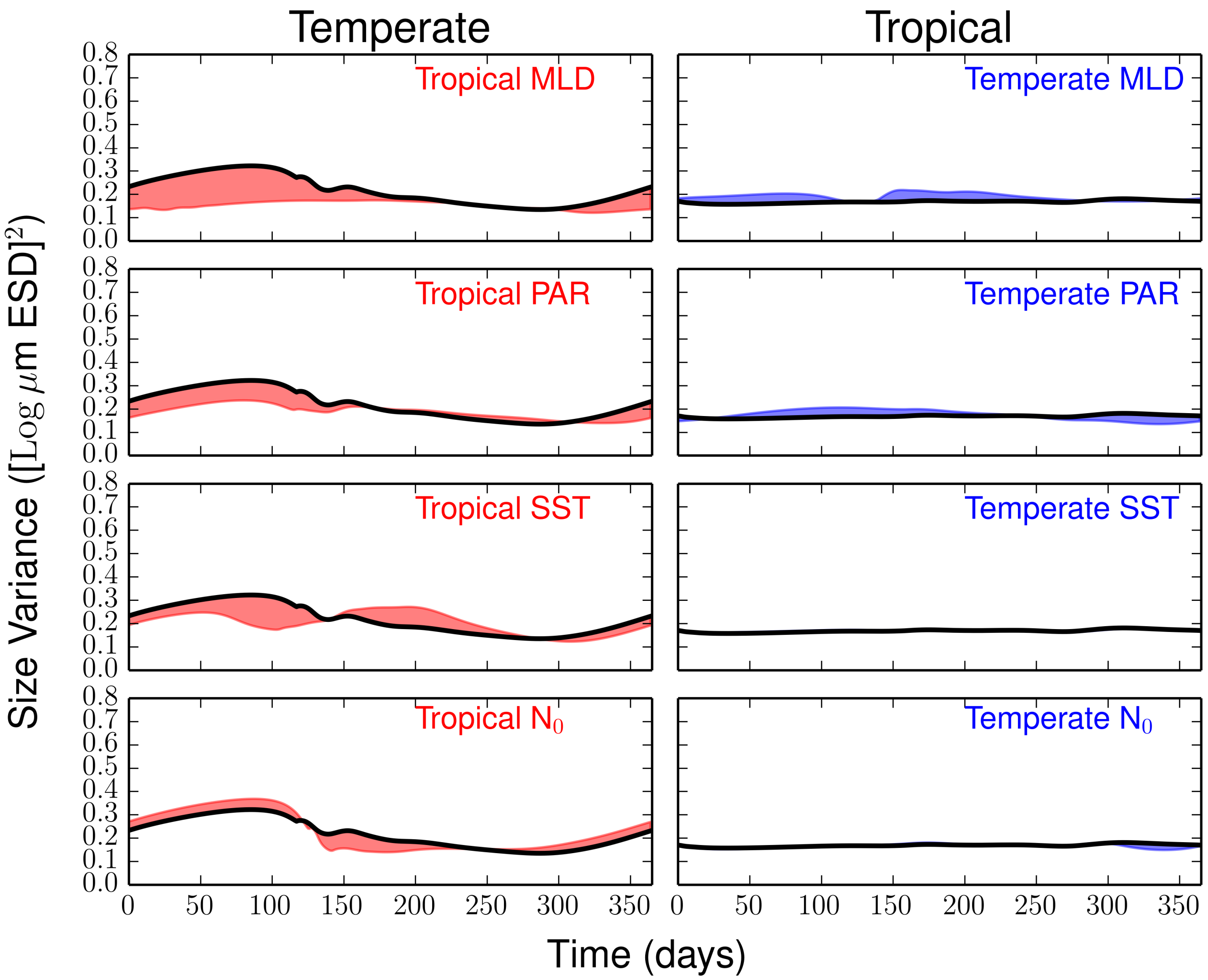

Supplementary Figure S5. Sensitivity of functional diversity (expressed by the size variance) to changes in environmental forcing. The black lines are the diversities obtained with the standard runs. The red areas in the temperate setup (left panels) show the effects on the diversity in the temperate region when one of its environmental conditions is changed with one of the tropical region. The blue areas in the tropical setup (right panels) show the effects on the diversity in the tropical region when one of its environmental conditions is changed with one of the temperate region.

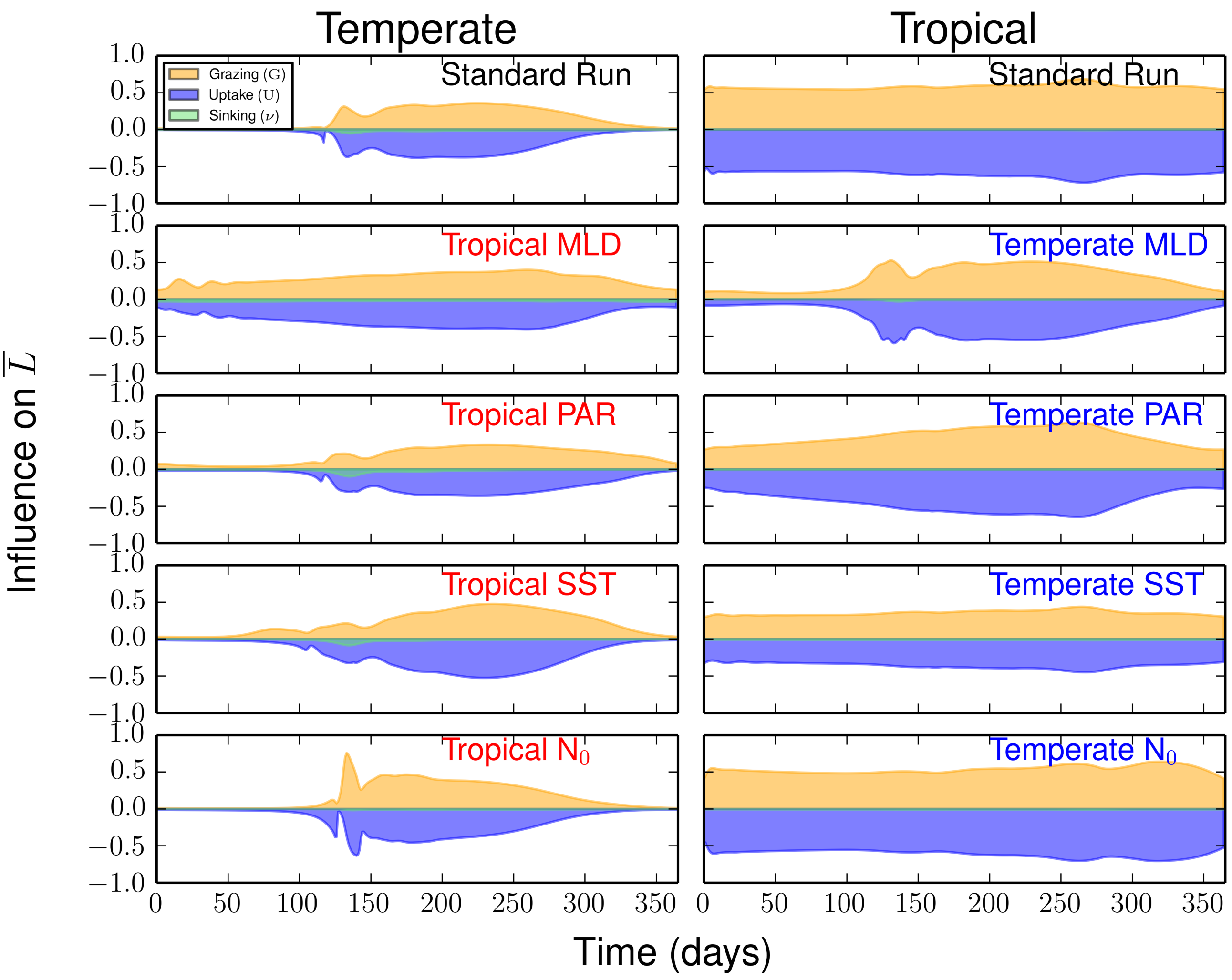

Supplementary Figure S6. Sensitivity of the size-scaling processes to the environmental forcing. The orange area represents zooplankton grazing (Equation 12), the blue area represents nutrient uptake (Equation 11), and the green area represents phytoplankton sinking (Equation 13). The left panels show the effect on the size-scaling processes in the temperate setup when one of the forcing is substituted with the corresponding forcing of the tropical setup. The right panels show the effect on the size-scaling processes in the tropical setup when one of the forcing is substituted with the corresponding forcing of the temperate setup.

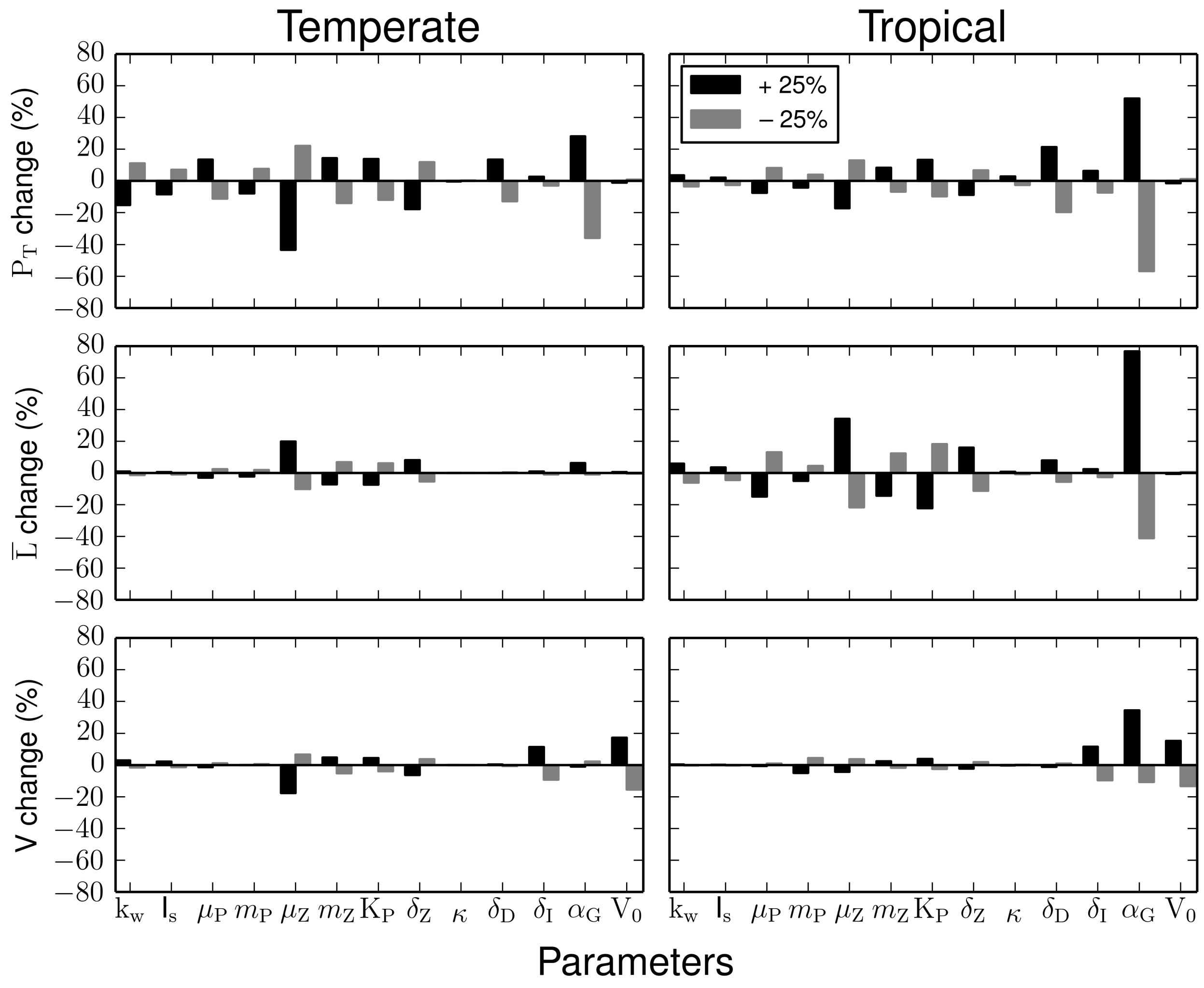

Supplementary Figure S7. Sensitivity analysis of the three macroscopic properties (P, $\overline{L}$, and V) to $\pm 25\,\%$ changes in parameter values. All parameters are listed in Table 1.

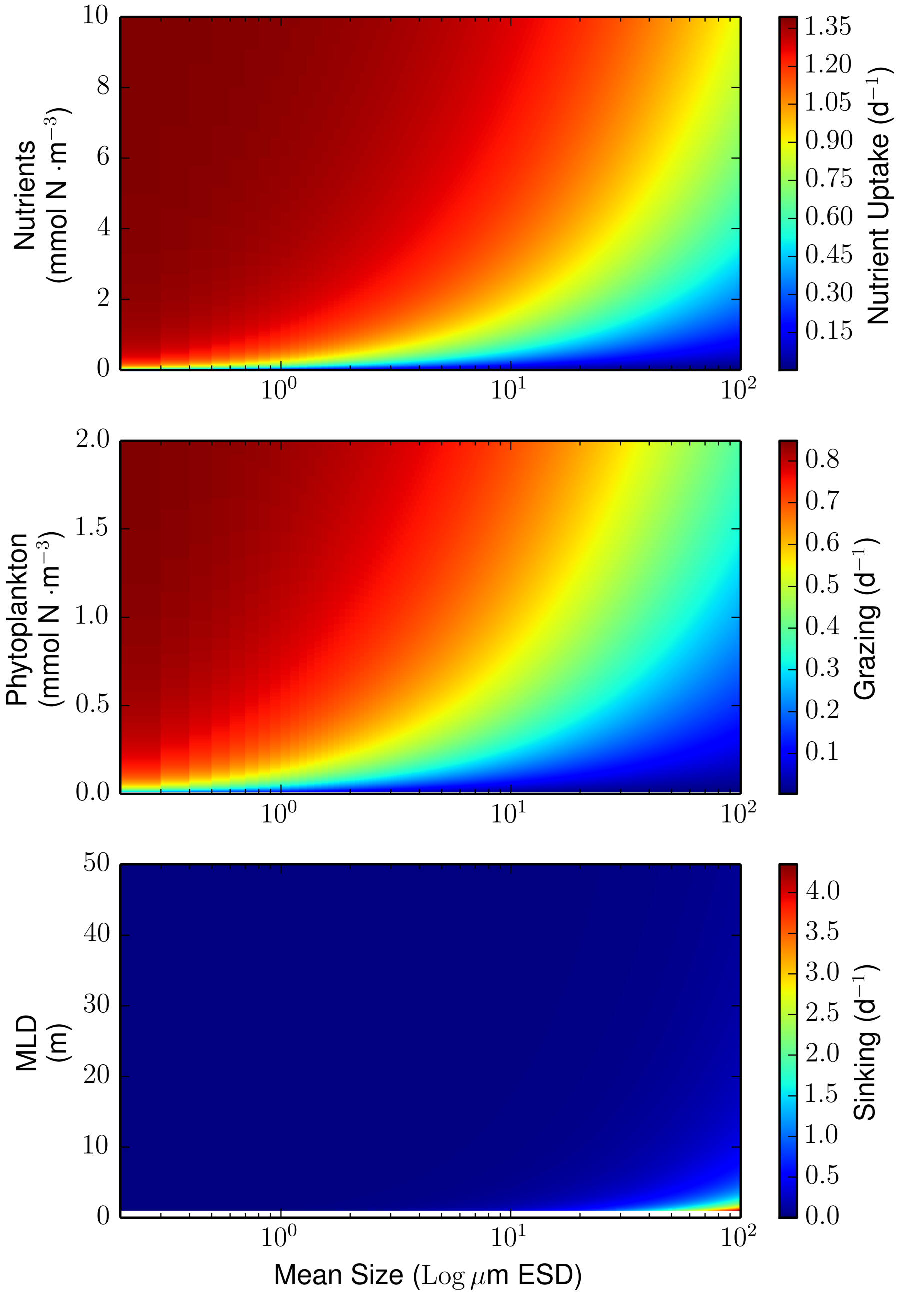

Supplementary Figure S8. Size-scaling processes. The panels show the relationship between phytoplankton cell size and A) nutrient uptake (Equation 11), B) zooplankton grazing (Equation 12), and C) phytoplankton sinking (Equation 13).

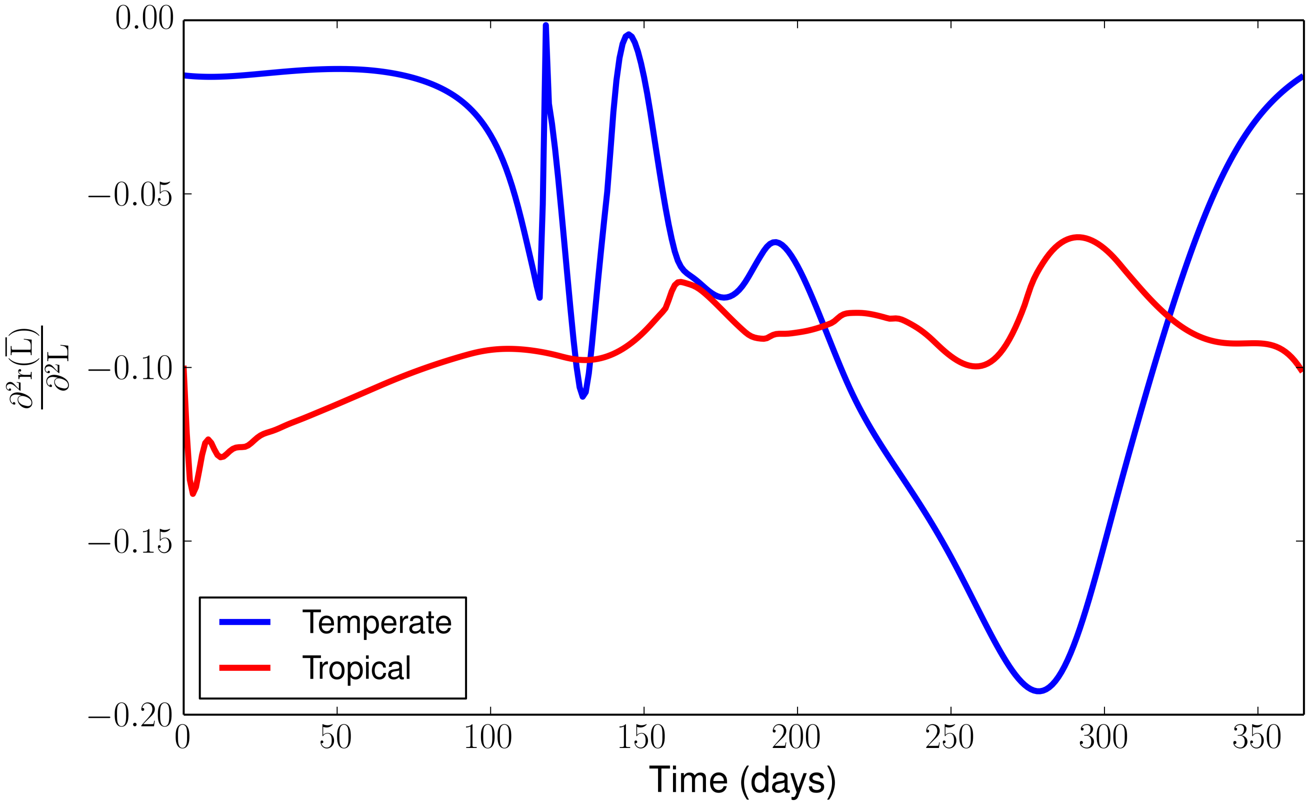

Supplementary Figure S9. Second derivative of the phytoplankton growth rate r with respect to logarithmic cell size L over the course of a year for the temperate (blue) and tropical region (red). This term determines the rate of change of the size variance in the system, with lower values indicating a faster decrease in variance. Consistently negative values indicate that there is no inherent source of variance in the systems.
